# Quantification and Ecological Risk Assessment of Colloidal Fullerenes Nanoparticles in Sediments by Ultrasonic-Assisted Pressurized Liquid Extraction and High Performance Liquid Chromatography

**DOI:** 10.3390/nano11123319

**Published:** 2021-12-07

**Authors:** Nokwanda Hendricks, Olatunde Stephen Olatunji, Bhekumuzi Prince Gumbi

**Affiliations:** School of Chemistry and Physics, University of KwaZulu-Natal, Durban 4001, South Africa; 215044619@stu.ukzn.ac.za (N.H.); olatunjio@ukzn.ac.za (O.S.O.)

**Keywords:** nanoparticles, fullerene, ultrasonic-assisted pressurized extraction, chromatography, nanomaterials, organic nanoparticles, ecological risk assessment

## Abstract

Fullerenes engineered nanomaterials are regarded as emerging environmental contaminants. This is as their widespread application in many consumer products, as well as natural release, increases their environmental concentration. In this work, an ultrasonic-assisted pressurized liquid extraction (UAPLE) method followed by high performance liquid chromatography with ultraviolet-visible detector (HPLC-UV-vis) was developed for extraction and determination of fullerene in sediments. The method was validated and found to be suitable for environmental risk assessment. Thereafter, the method was used for the determination of fullerene (C61-PCBM) in sediment samples collected from Umgeni River, South Africa. The current method allows for adequate sensitivity within the linear range of 0.01–4 µg g^−1^, method limit detection of 0.0094 µg g^−1^ and recoveries ranged between 67–84%. All the parameters were determined from fortified sediments samples. The measured environmental concentration (MEC) of fullerene in the sediment samples ranged from not detected to 30.55 µg g^−1^. To the best of our knowledge, this is the first report on the occurrence and ecological risk assessment of carbonaceous fullerene nanoparticles in African sediments and biosolids.

## 1. Introduction

Engineered nanomaterials including fullerenes have found broad applications in nearly all consumable and hardware products globally, due to their ready availability and affordability. Fullerenes belong to the group of carbonaceous nanomaterials. Consequently, residues of fullerene emanating from the use and application of fullerene containing products have led to their presence in the environment [1]. Although the fate and ecological risk fullerene pose to the environment is not clearly understood, has been reported that fullerenes containing the C60 colloidal aggregates form could interact with biological systems in aqueous environment [2,3,4]. In addition, the toxicities of fullerenes suspensions and their derivatives in water is dependent on the mechanisms of biological interaction. The fate and effects of fullerenes and other engineered carbonaceous nanomaterials are therefore of emerging concerns, especially due to their suggested toxic and health risks to the environment [5].

The utilisation of fullerenes containing products increase the exposure of them in the environment. In the aqueous environment, fullerenes accumulate in sediments, thereby exposing sediment resident microorganisms [6,7,8]. The eco-toxicology effects of fullerenes on aquatic organisms are not clearly characterised, as the methods to investigate possible toxicological effect of carbonaceous nanomaterials are currently under development [9,10,11]. Nybom et al. [6] studied the exposure of *Chironomus riparius* to fullerene-contaminated sediments, and suggested the induction of oxidative stress on these invertebrates, although they were localised in tissues under their microvilli layer [12,13]. Additionally, Torre et al. [14] reported the interaction of fullerenes with other environmental contaminants and noted that bioavailability and toxicity of fullerene on zebrafish embryos was enhanced by its association and interaction with benzo-pyrine [15,16,17].

Natural sediments have complex characteristics, which complicate efficient extraction of fullerene and other low concentration levels of emerging contaminants. A number of studies have reported the development of extraction methods for the recovery of fullerenes from soil and sediments, and these are based on soxhlet extraction and sonication assisted extraction. Soxhlet extraction method uses a larger volume of extraction solvent, hence it has hardly been reported or employed for the extraction of fullerene in recent times [18,19,20,21,22]. To study the fate and occurrence of fullerenes in the environment, sensitive and robust analytical methods need to be developed [23]. Wang et al. [24] developed dispersive liquid–liquid extraction method that uses less solvent, and can be applied to study fullerenes in the environment, using HPLC-UV-vis as the detection method. Sanchis et al. [25] developed an ultrasound-assisted toluene extraction followed by liquid chromatography-mass spectrometry method to study the occurrence of fullerene in the Sava Rivers, an important tributary of the Danube River [9,26,27,28]. The occurrence of functionalized fullerenes in soils collected from agricultural field, analysed using a centrifuge, sonication, shaker, and toluene as a solvent, followed by HPLC-UV-Vis detection, was also reported by Carboni et al. [29]. In a study by Shareef et al. [30], fullerene of up to 20 µg kg^−1^ in soils was quantified using HPLC-UV after extraction with accelerated solvent extraction. A sensitive method for detection of pristine fullerene nanomaterials in sediments using HPLC-UV-Vis developed by Perez et al. [31] had a detection limit of 0.9 ng g^−1^, which compares (equivalent) with that of mass spectrometer detection [27,28,32].

Regardless of the progress on the development of the analytical methods for the analysis of fullerenes nanomaterials, their investigation in the environment is still limited. Furthermore, most available studies in the literature reported the occurrence of pristine fullerenes (not the engineered functionalized form). HPLC-UV-Vis has so far been the technique of choice for assessing the occurrence of fullerenes in complex environmental matrices; however, their recovery from different environmental matrices is still a challenge [24,28,29,30,31,32,33]. In order to be able to study the occurrence and fate of fullerenes in sediment and the environment in general, a simple, sensitive, rapid, and robust analytical method needs to be developed [23].

The main objective of the present study was to develop a cheap, sensitive, and fast method for extraction of functionalized fullerene in sediments or biosolids systems of African environments by UAPLE. The effect of the extraction solvent volume, sonication time, and extraction solvents on the extraction efficiency of the method was studied. The method was validated and applied to extract sediment samples, collected from Umgeni River system, and biosolid from wastewater treatment plants. According to the authors’ knowledge, this is the first report on the determination of the occurrence of engineered functionalized fullerene nanomaterials in African sediments, using UAPLE as a fullerene extraction method. Ecological risk assessment of fullerene in sediments from values obtained experimentally measured environmental concentration (MEC) and highest observed no effect concentration (HONEC) is a first report, as the HONEC of C61-PCBM was recently established by Ponte et al. [10] in 2019 in toxicology studies of organisms in sediments.

## 2. Materials and Methods

### 2.1. Chemicals and Reagents

Fullerene standard C60 fullerene, [6,6]-phenyl C61 butyric acid methyl ester (Abbreviation. C61-PCBM > 99.5% purity, was purchased from Sigma-Aldrich (Steinheim, Germany). All solvents used, including toluene, acetone, ethyl acetate, acetonitrile, ethanol, and methanol, were chromatographic grade solvents (99%) and were Sigma Aldrich (Steinheim, Germany) products supplied by Merck (Darmstadt, Germany).

### 2.2. Apparatus and Glassware

Glassware such as glass beakers were washed, rinsed with acetone, and heated at 150 °C for 24 hr to prevent emulsion. Micropipettes (plus kit Dragon lab (China)) ranging from 100 μL, 1000 μL, and 5 mL, were used for measurements of small volumes. Solid phase extraction vacuum manifold (24 positions) was bought from Separations, South Africa. All 0.45 μm glass fibre Millipore filters were purchased from Anatech, South Africa. Aluminium foil, syringes used as separating columns, and mortar and pestle used for homogenising the sediments samples were bought from university store. Chromatography vials were bought from Chemetrix, South Africa. A metal shovel used for sediment sample scooping was bought from Makro South Africa. Sieves (mesh sizes 54–600 µm) used to sieve sediment samples after grinding, was bought from KingTest laboratory, South Africa.

### 2.3. Instruments

The identification and the quantification of the analyte was conducted with a HPLC Agilent (1200 series) equipped with an automatic injector and coupled to a 1100 series MSD Trap UV-vis detector (Agilent Technologies, Santa Clara, CA, USA). To measure nanoparticles size and shape, high-resolution transmission electron microscopy (HRTEM) was used, ultrasonic bath (Scientech ultrasonic cleaner; frequency 50 kHz, and a nominal power 150 W) bought from Anatech was used to accelerate the extraction, and portable pH meter (Thermo Scientific, Eutech, Singapore), was used to measure environmental quality parameters such as pH, conductivity, and TDS. Hot air oven (50–150 °C) was used to dry all the glassware used. Unless stated, all instruments were bought from South Africa.

The elution was undertaken on C18 Gemini-NX 3u (150 × 2.1 mm) column with toluene (100%) as mobile phase. The HPLC was operated on an isocratic mode at a flow rate of 0.400 mL min^−1^. The sample injection volume was 2 µL, and the wavelength selected for detection and quantification of fullerene (C61-PCBM) was 338 nm.

### 2.4. Sample Collection

**Figure 1:**
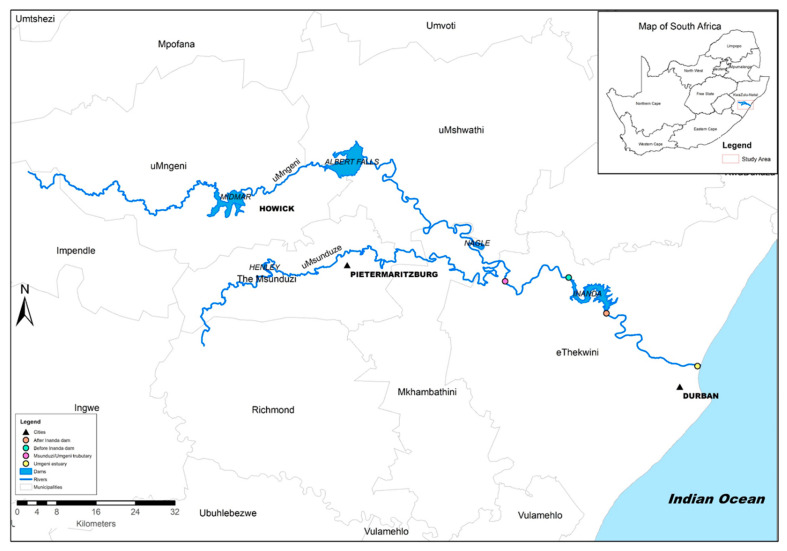
Map for the sediments sampling points.

Sediment samples were collected along Umgeni River, and biosolids or sludge were collected at two wastewater treatment plants located in KwaZulu-Natal, South Africa. Map of sampling sites are presented in Figure 1. This site is crucial as it is located downstream of wastewater treatment plants that practice indirect water reuse. After the treatment process, these plants discharge effluent into Mgeni River for potable use downstream, and this practice relies on natural processes to rehabilitate contaminated water. As nanomaterial agglomerates in surface waters and settles into river sediments, there is need to investigate the ecological risk of river ecosystem that are situated downstream of water treatment plants. Moreover, an ecological risk assessment is paramount, as the greater city of Durban relies on these waters for consumption. The samples were scooped with a metal shovel in the upper 10 cm of sediment, wrapped with aluminium foil, covered with a plastic zip bag, and well labelled. Biosolids or sludge samples were collected in the wastewater treatment plants separation grid, wrapped with aluminium foil, placed in the plastic bags, and thereafter labelled. All samples were transported to the laboratory in cooler boxes packed with ice to preserved sample at temperatures under 4 °C.

### 2.5. Sample Preparation

The samples were dried at ambient temperature for 48 h, thereafter homogenised with mortar and pestle, and then sieved using a sieve with particle mesh size < 54 µm. The sieved samples were stored in a glass container covered with foil and kept in a dry and cool environment until further analysis.

### 2.6. Preparation of Fullerene Solutions

#### Preparation of Fullerene Working Standard Solution, Spiked Sediment, and Sample Extraction

Firstly, 100 mg L^−1^ stock solution of fullerene was prepared by dissolving 10 mg C61-PCBM in 100 mL of toluene in a volumetric flask. The working stock solutions of 10 mg L^−1^ was prepared by transferring 100 µL of 100 mg L^−1^ into 1 mL volumetric flask and made up to mark with toluene. Furthermore, working solution of 1 mg L^−1^ was prepared by transferring 100 µL of 10 mg L^−1^ into 1 mL volumetric flask and made up to mark with toluene. All solutions were transferred with a micropipette. All the working standards for external calibration were prepared by consecutive dilution using toluene as dilution solvent. The concentration of the working standards prepared from 100 mg L^−1^ were 0.1 mg L^−1^, 1 mg L^−1^, 3 mg L^−1^, 5 mg L^−1^, 10 mg L^−1^, 20 mg L^−1^, 30 mg L^−1^, and 40 mg L^−1^. The 0.1 mg L^−1^, 1 mg L^−1^, and 3 mg L^−1^ working solutions were prepared from the 1 mg L^−1^, 10 mg L^−1^, and 30 mg L^−1^ solutions, respectively. The calibration curve was constructed using these standards range.

A portion of the sieved sediment samples was proportionally mixed and washed with toluene to make the matrix uniform by removing analytes in the matrix. To prepare matric matched internal calibration curve, 10 g of washed sediments was weighed into a beaker, then spiked with C61-PCBM prepared in toluene as per the prepared working standards. To ensure the distribution of fullerene in sediments after spiking, 10 mL toluene contain C61-PCBM was added to the 10 g sediments in the 50 mL beaker. The spiked samples were left in the dark overnight to dry, and thereafter the dried sediments were quantitatively transferred from the beaker into 50 mL syringes vessel set-up equipped with a 0.45 µm syringe filter as a stopper. Then, into the syringe set-up, 8 mL toluene was added as an extraction solvent and sonicated for 15 min. After sonication, syringes containing samples were placed in the manifold and vacuum extracted. This was performed in two cycles; the extracts were combined and left overnight in the dark to pre-concentrate to 200–500 µL. The pre-concentrated extracts were transferred into vials and made up to 1 mL constant volumes. The samples were then analysed with HPLC-UV-vis. The signal (peak areas) obtained at each concentration levels were used to plot the internal calibration curve. The samples extraction was also performed in a similar way.

### 2.7. Limit of Detection and Limit of Quantification Calculations

The method’s limit of detection (MLOD) and method’s limit of quantification (MLOQ), were determined using an independent external standard, prepared by fortifying sediments and analysed under optimum conditions. For quality purposes, all analysis were performed in triplicate, error bars are presented in graphs, and in table standard deviations are presented.

(1) $$\mathrm{MLOD}=3\times \frac{\sigma \;}{S}$$

(2) $$\mathrm{MLOQ}=10\times \frac{\sigma }{S}$$

where σ is the standard deviation triplicate and s is the slope of the calibration curves.

### 2.8. Ecological Risk Calculation

In many previous studies, the risk is calculated from predicted environmental concentration (PEC) and predicted no effect concentration (PNEC) established from probabilistic species sensitivity distributions (pSSDs) values [10,34,35,36,37,38]. However, in this study, the risk characterisation ratio (RCR), normally referred to as hazard Risk Quotient (RQ), was calculated based on the established formula, but using measured environmental concentration (MEC) with the proposed extraction method and highest observed no effect concentration (HONEC) taken from toxicological studies of Ponte et al. [10] to assess C61-PCBM ecological risk in studied river system:

(3) $$\mathrm{RCR}=\frac{\mathrm{MEC}}{\mathrm{HONEC}}$$

Therefore, an RCR < 0.1 would indicate that ecological risk is low for this studied environment. An RCR between 0.1 and 1 would indicate that ecological risk is medium for this studied river system. An RCR > 1 would mean the measured environmental concentrations are high enough to negatively impact the organisms living in these river sediment and if RCR is above 1, there is a need to introduce risk management measures to mitigate the environmental impact. This approach is relevant in assessing the ecological risk posed by C61-PCBM in environmental organisms, as values used in the formula were found experimental in realistic sediments conditions.

## 3. Results

### 3.1. Ultrasonic-Assisted Pressurized Liquid Extraction Method Development

Parameters such as extraction solvent, the volume of extraction solvent, and sonication time were investigated and were optimised in order to improve the performance of the method.

#### 3.1.1. Selection of Extraction Solvent

**Figure 2:**
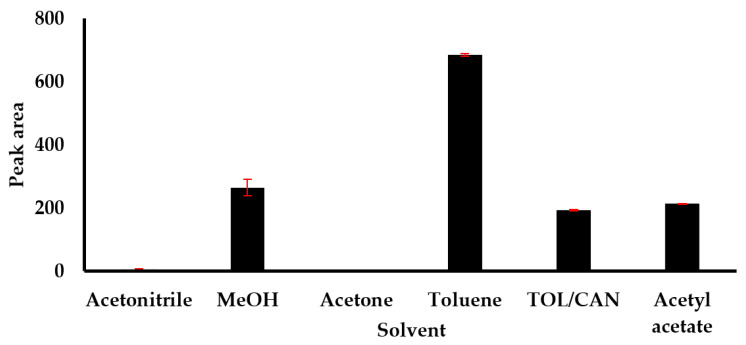
Efficiency of extraction solvent in the recovery of fullerenes.

The selection of suitable extraction solvent of nanoparticles from environmental matrices, such as sediments and biosolids, is an intricate task as these matrices contain a number of contaminants that might interfere with their detection and quantification. The choice of solvent is important to ensure high recoveries of nanoparticles while preventing the extraction of undesired contaminants from the matrix for better detection. Various solvents were selected to cover a wide range from hydrophobic to polar, including toluene, acetonitrile, methanol, acetone, and acetyl acetate with the constant volume of 8 mL and sonication time of 15 min. The efficiency of different solvents for extraction of fullerene from sediments or biosolid presented is shown in Figure 2.

Signal from the analysis of fullerene in toluene extract was the highest, indicating good recovery using toluene as extraction solvent. This could be due to high solubility of C61-PCBM in toluene compared to other organic solvents investigated in this study, and thus toluene was selected as the extraction solvent. Moreover, chromatographic method uses toluene as a mobile phase, which make it easier to reconstitute when toluene is an extraction solvent to avoid enhancement of signal.

#### 3.1.2. Selection of Toluene Volume

**Figure 3:**
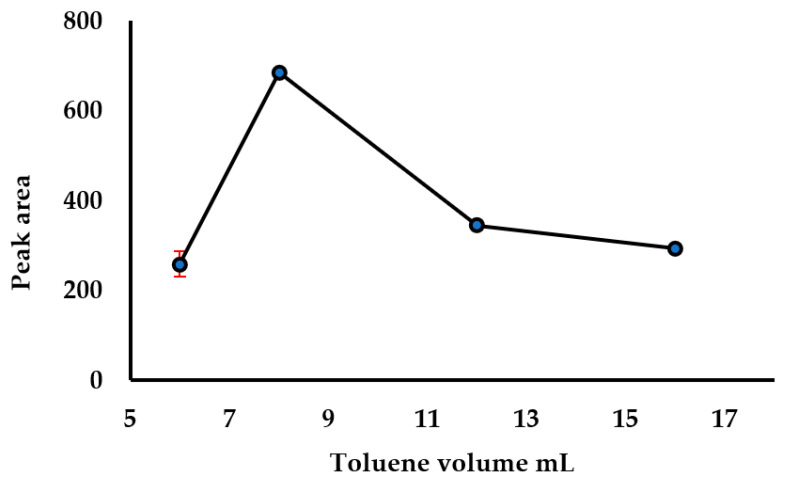
Effect of toluene volume in extraction efficiency.

The previously developed methods for the analysis of fullerene in environmental matrices makes use of a larger amount of solvent (toluene) in the extraction process, ranging from 25–100 mL [31]. However, lower extraction volumes are usually desired, as toluene is not an environmentally friendly solvent, even though it is a good extraction solvent for fullerenes. Therefore, toluene volume (6–16 mL) as the extraction solvent was evaluated. The results showed an increase in extraction efficiency when the toluene volume increased from 6–8 mL, and the efficiency decreased above 8 mL (Figure 3). The 8 mL was chosen as the optimal solvent volume. A solvent extraction volume lower than 6 mL was found to be not enough to entirely wet the 10 g sample.

#### 3.1.3. Evaluation of Sonication Time

**Figure 4:**
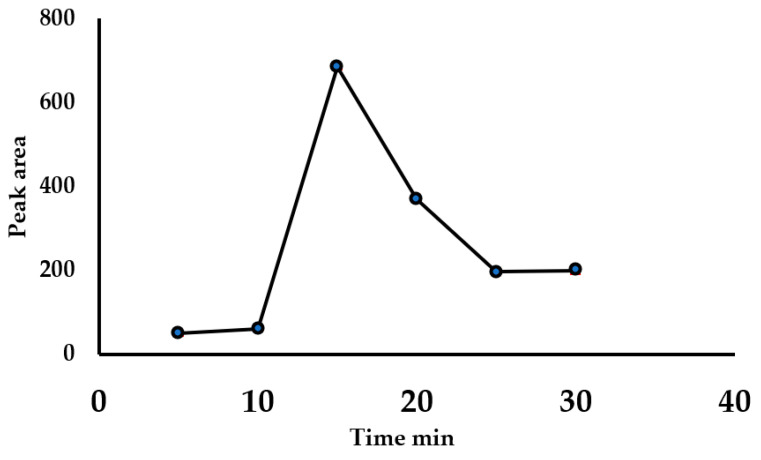
Effect of sonication time during extraction.

To evaluate the sonication time, six sediment samples that were previously washed with toluene were spiked with 1 µg g^−1^ C61-PCBM. All other extraction parameters were kept constant, while the resident time of sonication was varied from 5–30 min. There was a slight increase in the extraction efficiency when the time was increased from 5–10 min (Figure 4). This was attributed to the fact that the 10 min was not sufficient to allow the solvent to penetrate the sediment lattice and in order to free and disperse fullerene into the toluene phase during sonication. An exponential increase in the extraction of fullerene into toluene between 10 min and 15 min was observed, which was followed by a sharp decrease after 15 min.

Sonication time did not affect the extraction efficiency after 25 min. Hence, the optimum extraction time of fullerene from sediment using toluene as extraction solvent in ultrasonic assisted liquid extraction was observed at 15 min.

### 3.2. Method Validation

The performance of the ultrasonic assisted liquid extraction method was evaluated using optimised UAPLE conditions, with toluene as an extraction solvent, an extraction volume of 8 mL in two cycles (total volume of 16 mL), and a 15 min sonication time. The extracted samples were analysed using HPLC-UV-vis. The method was validated for linearity, linear range, recoveries, limit of detection and limit of quantification, precision, and accuracy [39].

#### 3.2.1. Linearity

**Figure 5:**
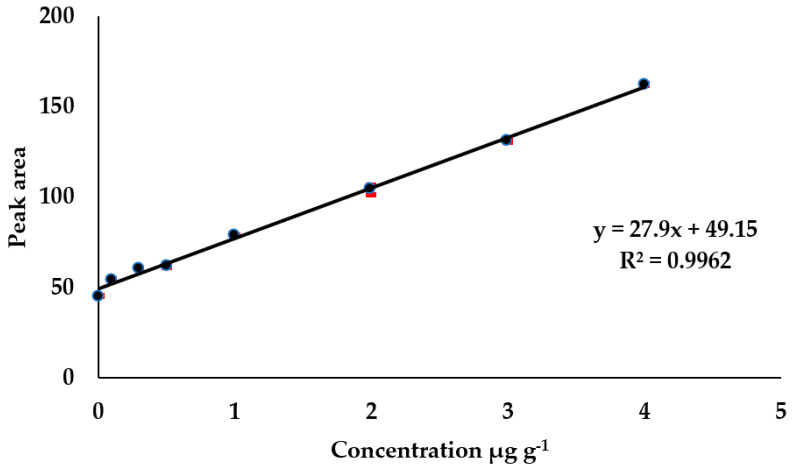
Calibration curve of peak area against C61-PCBM concentration in sediments; a matrix matched calibration curve.

The response of analytical method to concentration of fullerene in toluene extracts was assessed by examining the linearity of signal generated with increasing gradient concentrations of fullerene in toluene extracts. Sequential response of eight concentration levels of fullerene, with linearity ranging from 0.01–4 µg g^−1^, was evaluated. The calibration curve was based on matrix matched, established by plotting peak area (signal) against concentration as shown in Figure 5. A linear relationship was observed in terms of the analysis response to increasing concentration. The equation for the best fit was y = 27.9x + 49.15 with coefficient of linear regression 0.9962.

#### 3.2.2. Limit of Detection and Limit of Quantification and Recoveries

The MLOD was defined for a signal-to-noise ratio of 3 and found to be 0.0094 µg g^−1^, as per the MLOD equation below, while the LOQ defined for a signal-to-noise ratio of 10 was 0.031 µg g^−1^, as per the MLOQ equations below.

The observed MLOD and MLOQ were in range consistent with other methods previously developed for the analysis of fullerenes in sediments [9,27,32,40].

The environmental suitability of the extraction method was determined by fortifying the studied matrix in two concentration levels, 1 µg g^−1^ and 4 µg g^−1^. The recoveries ranged between 67% and 84% in all the studied different concentrations levels, as shown in Table 1. The established range was in line with IUPAC guidelines stated by Thomson et al. [39].

#### 3.2.3. Precision and Accuracy

The precision of the method expressed as percentage relative standard deviation (%RSD) was evaluated by extracting 12 samples in consecutive days fortified at two concentration levels of 1 µg g^−1^ and 4 µg g^−1^ to cover the calibration curve extremes. Inter- and intra-day precision ranged from 0.44–0.67% and 0.27–0.40%, respectively, as shown in Table 2.

The accuracy of the entire method was evaluated by fortifying two independent sediments with 1 µg g^−1^ and 4 µg g^−1^ fullerene. The samples were processed and analysed using the developed method. Their concentrations were determined from the matrix matched calibration curve. To determine the accuracy of the entire method, two independent fortified sediments with the known concentration of 1 µg g^−1^ and 4 µg g^−1^. The accuracy was established to be between 99% and 108% with percentage errors of −1% and 8%, respectively.

These results are within range of other fullerenes methods used for fullerenes [24,29,30,31]. The overall validation results allowed the method to be used in environmental detection and analysis of fullerenes in wastewater treatment plants.

## 4. Discussion

### 4.1. Environmental Analysis Fullerene

**Figure 6:**
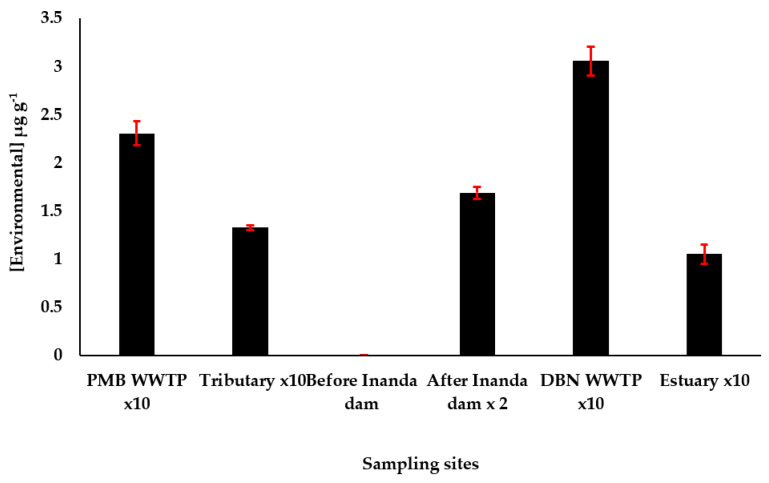
Environmental sediment samples analysis using developed analytical method.

The validated UAPLE was applied in the determination of fullerene (C61-PCBM) in sediment samples from four river sites and two wastewater treatment plants biosolids or sludge samples. The results of the analysis of fullerene are presented in Figure 6.

The C61-PCBM was not detected in sediment samples collected from the sampling site before the Inanda Dam. This could be as the sediment sample collected at this sampling site is sandy with low retention capacity for fullerene, hence the C61-PCBM might have leached below the surface sediment, as the samples were taken in the 10 cm of the upper sediment surface.

However, C61-PCBM was found in high concentration levels outside the linearity of the calibration curve in sediments from other sampling sites. Consequently, sample extracts were first diluted in 2 mL toluene, and then further diluted to 10 mL in order to fall within the methods calibration curve so as to find the environmental concentration. The highest concentration of 30.55 µg g^−1^ was found in Durban Wastewater Treatment plant (DBN WWTP), while the lowest concentration of 10.52 µg g^−1^ was found in Mgeni Estuary (Estuary) as shown in Figure 6. The detection of fullerene in higher concentration is attributed to broad usage of these compounds in commercial products, household and industrial applications, and usage in the wastewater treatment plant for reclamation of wastewater. The detection of fullerene in tributary sediments may be associated with natural or wildfire phenomena, as fullerenes can be found naturally or during wildfires [40].

Many researchers have reported the presence of fullerene (C61-PCBM) in the environment [22,25,32]. Most of the developed analytical methods used for analysis of fullerenes in the environment are based on HPLC-UV-Vis, as shown in Table 3. Some of the developed analytical methods were not applied in the analysis of real environmental samples, but rather simulate contaminated sediments or artificial sediment, as opposed to the developed methods.

The volume of extraction solvent affects extraction efficiency; Table 3 shows high volume leads to better recoveries. However, this is also depends on the sample mass used. In the current method, a low volume of extraction, the solvent used was compensated by combining the ultrasonication and pressurized techniques to improve the recoveries [29]. The current method detection limit and recoveries were in range with other previous studies, as depicted in Table 3.

### 4.2. Ecological Risk Assessment of Fullerenes in Mgeni System Sediments

In the environment, nanomaterials agglomerate, which leads to their sedimentation. Therefore, assessment of ecological risk of organisms exposed to nanomaterial-containing sediments is paramount [38,41]. Additionally, ecological risk assessment studies must focus on crucial biological species that are directly or indirectly responsible for ecosystem processes in the environment, such as benthinic organisms [28]. These organisms drive numerous ecosystem functions in the environment. Moreover, many risk assessment agencies recommend usage of data found using crucial biological species, such that the obtained data may be used in other risk assessment studies to prevent further usage of organisms. Hence, in this work, the HONEC value of 25 mg kg^−1^ of C61-PCBM used in PCR calculation was established by Ponte et al. [10] from benthinic organisms in realistic sediment conditions. In addition, Coll et al. [41] found the same value 25 mg kg^−1^ of C61-PCBM in soil in a separate study. This value, together with MECs determined using our developed method, enable the ecological risk of the studied area; the results are shown in Table 4. In all the sampling sites, risk characterization values ranged from 0.42 to 1.22, which indicate medium risk and high risk, respectively. In DBN WWTP, where high risk was established, this site requires further investigation, with the potential of developing guidelines for fullerenes levels in the effluent. The other site exhibited suspected medium risk; however, the sites of concern were the PMB WWTP and after the Inanda Dam, where risk characterization values were close to one, indicating suspected ecological impact. It is important to have accurate ecological data accompanied by informative environmental remediation or guidelines response, to improve risk assessment prediction tools; recommendations are outlined in the section below.

### 4.3. Further Recommendations

There is need to refine HONEC values to better predict ecological risk with respect to long-term exposure of fullerene into organisms. Chronic effect studies based on a clearly defined mode of action of fullerene onto organisms would be necessary to improve risk assessment methods and validate risk characterization values as early warning indicators for environmental protection. In this study, wastewater treatment plants were flagged as sites that need further investigation to determine the extent of the ecological impact of discharging effluent to rivers. Further research using mammals is recommended to evaluate these suspected high risk sites; it is anticipated that the animal studies would be targeted to the sites that showed medium to high risk, such as PMB WWTP, DBN WWTP, and after the Inanda Dam. This study indicates that there is a need for biosolids to be treated before they can be applied in soil amendment, which is a normal practise in wastewater treatment plants.

## 5. Conclusions

In this work, a new extraction method was developed and optimised for the detection and quantification of functionalised fullerene C61-PCBM in sediments using HPLC-UV-vis. The method was validated; the method limit of detection and method limit of quantification were 0.0094 µg g^−1^ and 0.031 µg g^−1^, respectively. The analysis of C61-PCBM extracted from real sediments samples fortified shows that the method is robust and suitable for detection of the fullerene in complex environmental matrices at a precision range of 0.27% to 0.67% and accuracy of 99% to 108%. The extraction of the C61-PCBM with the combination of ultra-sonication and pressurization in two cycles with toluene as an extracting solvent was highly repeatable and relatively efficient, with recoveries ranging from 67% to 84%. The method was applied in real environmental samples; C61-PCBM was detected in five out of six sampling sites. This is the first time C61-PCBM fullerene has been detected in sediments on the African continent. The method allowed the ecological risk of C61-PCBM to be assessed in sediment and be found to range from high to no risk. It will also help to expand the database of fullerene nanomaterials in the environment.

## Figures and Tables

**Table 1:** Validation data for the detection method of C61-PCBM in sediments.

Parameters	Validation Results
Linearity	0.01–4 µg g^−1^
Method detection limit	0.0094 µg g^−1^
Method quantification limit	0.031 µg g^−1^
Linear regression	0.9962
Recoveries	67–84%

**Table 2:** C61-PCBM recoveries and instrument repeatability.

	**Precision**	
	1 µg g^−1^	4 µg g^−1^
Inter-day% RSD	0.67	0.44
Intra-day% RSD	0.40	0.27
	**Accuracy**	
Fortified concentration	1 µg g^−1^	4 µg g^−1^
Calculated concentration µg g^−1^	1.08 ± 0.01	3.96 ± 0.03
Accuracy (%)	108	99
% Error	8%	−1%

**Table 3:** Comparison of analytical methods from literature with current method.

Instrument	Analyte	Extraction Method	Extraction Solvent	Extraction Volume	Sample Mass	Detection Limit	Recovery	Environmental Concentration	Reference
HPLC-UV	C60 & C70	Ultrasound-assisted extraction	Toluene	4 mL	5 g	0.9 ng g^−1^	72–104%	Not detected	Perez et al. [31]
HPLC-UV	C60	Accelerated solvent extraction	Toluene	40 mL	5 g	20 ng g^−1^	84–107%	-	Shareef et al. [30]
HPLC-UV	C60, C70, C61 & C71	Sonication & shaking extraction	Toluene	10 mL	10 g	3 ng g^−1^	47–71%	-	Carboni et al. [29]
HPLC-UV	C60	Shaking extraction	Toluene	40 mL	10 g	1500 ng g^−1^	83–108%	-	Wang et al. [24]
HPLC-UV	C61	Ultrasonic-assisted pressurized liquid extraction	Toluene	8 mL	10 g	9 ng g^−1^	67–84%	Not detected—30 µg g^−1^	Current method

**Table 4:** Ecological risk assessment of C61-PCBM in sediments from MEC and HONEC obtained realistic conditions.

Sampling Point	PMB WWTP	Tributary	Before Inanda Dam	After Inanda Dam	DBN WWTP	Estuary
MEC	23.05 ± 0.12	13.27 ± 0.02	-	16.87 ± 0.06	30.55 ± 0.15	10.52 ± 0.10
RCR	0.92	0.53	-	0.67	1.22	0.42
Ecological risk assessment	Medium risk	Medium risk	No risk	Medium risk	High risk	Medium risk

## Data Availability

Not applicable.
